# Decreased Expression of Beclin 1 Correlates Closely with Bcl-xL Expression and Poor Prognosis of Ovarian Carcinoma

**DOI:** 10.1371/journal.pone.0060516

**Published:** 2013-04-03

**Authors:** Huan-Xin Lin, Hui-Juan Qiu, Fei Zeng, Hui-Lan Rao, Guo-Fen Yang, Hsiang-Fu Kung, Xiao-Feng Zhu, Yi-Xin Zeng, Mu-Yan Cai, Dan Xie

**Affiliations:** 1 The State Key Laboratory of Oncology in South China, Sun Yat-Sen University Cancer Center, Guangzhou, China; 2 Department of Gynecology, The First Affiliated Hospital, Shantou University Medical College, Shantou, China; 3 Department of Pathology, Sun Yat-Sen University Cancer Center, Guangzhou, China; 4 Department of Gynecology, The First Affiliated Hospital, Sun Yat-Sen University, Guangzhou, China; 5 The State Key Laboratory of Oncology in South China, The Chinese University of Hong Kong, Hong Kong, China; The University of Hong Kong, China

## Abstract

**Background:**

It has been suggested that autophagy-related Beclin 1 plays a critical role in the regulation of tumor development and/or progression, but its prognostic significance and relationship with Bcl-xL expression in ovarian carcinoma are unclear.

**Methodology/Principal Findings:**

In the present study, the methods of Western blotting and immunohistochemistry (IHC) were utilized to investigate the expression status of Beclin 1 and Bcl-xL in fresh ovarian tissues and paraffin-embedded epithelial ovarian tumor tissues. Decreased expression of Beclin 1 was examined by IHC in 8.3% of normal ovaries, in 15.4% of cystadenomas, in 20.0% of borderline tumors, and in 55.6% of ovarian carcinomas, respectively. In ovarian carcinomas, decreased expression of Beclin 1 was correlated closely with ascending histological grade, later pT/pN/pM status and/or advanced clinical stage (*P*<0.05). In univariate survival analysis, a highly significant association between low-expressed Beclin 1 and shortened patient survival was evaluated in ovarian carcinoma patients (*P*<0.01), and Beclin 1 expression was an independent prognostic factor as evidenced by multivariate analysis (*P* = 0.013). In addition, decreased expression of Beclin 1 was inversely correlated with altered expression of Bcl-xL in ovarian carcinoma cohort, and combined analysis further showed that the low Beclin 1/high Bcl-xL group had the lowest survival rate.

**Conclusions/Significance:**

Our findings suggest that Beclin 1 expression, as examined by IHC, could be served as an additional tool in identifying ovarian carcinoma patients at risk of tumor progression, and predicting patient survival in ovarian carcinomas with increased expression of Bcl-xL.

## Introduction

Ovarian cancer is the most common cause of cancer death among gynecological malignancies worldwide, and the incidence has been steadily increasing in Asian countries such as China and Singapore [1], [2]. Ovarian carcinoma, which originates from ovarian surface epithelium, accounts for 90% of ovarian cancers in women [2]. The majority of patients with ovarian carcinoma were diagnosed at advanced stages due to absence of specific symptoms and lack of reliable methods for the early detection [3]. Thus, the long-term prognosis of patients with ovarian carcinoma remains poor despite recent progress in surgical techniques and chemotherapeutic treatments [4]. Recently, more and more advances have been made in understanding the genetic alterations and biological processes in ovarian carcinoma [5]. However, the search for specific molecular and/or genetic alterations in ovarian carcinoma that have clinicopathologic/prognostic significance is substantially limited.

As any other critical functions of cellular biology, autophagy of cellular proteins through an autophagosomic-lysosomal pathway, is significant in normal cell growth control and may be defective in tumor cells [6]. Beclin 1, the first identified mammalian autophagy gene product, has been mapped to a tumor-susceptibility locus on chromosome 17q21 and is a haploinsufficient tumor suppressor that was originally isolated as a Bcl-2-interacting protein [7]. The critical autophagic effects of Beclin 1 protein may be attributed to its interaction with several important cellular molecules, including Bcl-2 [8], [9], Bcl-xL [10], HIF-1 [11], mTOR [12], JNK [13], Ambra1 [14], Bif-1 [15], Vps34 [16], Atg14 and UVRAG [17]. Bcl-xL, an antiapoptotic protein from the Bcl-2 family, was initially characterized as cell death regulator and has been suggested to control the autophagic process recently [18], [19]. The deletion of the status of *Beclin 1* gene in human cancers may promote tumorigenesis, as targeted mutant mice with heterozygous disruption of the *Beclin 1* gene could decrease autophagic activity and spontaneously develop tumors including lung cancer, lymphoma, hepatocellular carcinoma and mammary precancerous lesions [20], [21]. Recently, Beclin 1 has been identified as a reliable biomarker in monitoring the prognosis for several tumors, such as brain, liver, gastric, colorectal, nasopharyngeal cancers and NK/T cell lymphoma [22], [23], [24], [25], [26]. Furthermore, we found that decreased expression of Beclin 1 was correlated with the development of epithelial ovarian tumors [27]. Nevertheless, the prognostic significance of Beclin 1 in ovarian carcinoma and its relationship with Bcl-xL expression have not been elucidated.

In the present study, we measured the expression levels of Beclin 1 and Bcl-xL by Western blotting and immunohistochemistry (IHC) in human epithelial ovarian tumors with normal ovarian tissues as controls. Meanwhile, X-tile software version 3.6.1 (Yale University School of Medicine, New Haven, CT), a reliable promising program to analyze optimal cutpoint of biomarkers [28], was introduced to determine the cutpoint of Beclin 1 and Bcl-xL IHC expressions in our ovarian carcinoma cohort and thus, the clinicopathologic/prognostic value of Beclin 1 expression and its association with Bcl-xL in ovarian carcinomas were analyzed. We now report that decreased expression of Beclin 1 is closely associated with a more aggressive phenotype and/or poor prognosis of ovarian carcinoma synergized with increased expression of Bcl-xL.

## Materials and Methods

### Ethics statement

The study was approved by the Institute Research Medical Ethics Committee of Sun Yat-Sen University. No informed consent (written or verbal) was obtained for use of retrospective tissue samples from the patients within this study, most of whom were deceased, since this was not deemed necessary by the Ethics Committee, who waived the need for consent. All samples were anonymised.

### Patients and tissue specimens

Formalin-fixed and paraffin-embedded tissue samples from 230 patients with epithelial ovarian tumors were obtained from archives of Department of Pathology, the First Affiliated Hospital, Sun Yat-Sen University, Guangzhou, China, between 1996 and 2008. The tumor cases included 169 cases with histologically confirmed invasive carcinoma, 35 cases with borderline tumors and 26 cases with benign cystadenoma. In addition, twelve normal ovaries from hysterectomy specimens resected for non-ovarian disease in our institute were added for IHC analysis. The cases selected were based on availability of resection tissue, follow-up data and those not having received preoperative treatment.

Ages of the 169 patients with ovarian carcinoma ranged from 19 to 84 years (mean age, 50.8 years) and the average duration of follow-up was 36.3 months (range, 0 to 143.0 months). Clinicopathologic features of this ovarian carcinoma cohort are described in Table 1. The stage of tumors was assessed according to the International Federation of Gynecology and Obstetrics (FIGO) system. Tumors were graded according to the Silverberg grading system. All the cancer cases were reevaluated for grade and histological type by the senior pathologists (M.-Y. C. and H.-L. R.). Moreover, for Western blotting, fresh tissue specimens from 5 primary ovarian carcinomas and corresponding adjacent normal ovaries underwent surgical resection were collected in 2009 in our institute. The Institute Research Medical Ethics Committee of Sun Yat-sen University granted approval for this study.

**Table 1:** Association of Beclin 1 Expression with Patients' Clinicopathologic Features in Ovarian Carcinomas.

		Beclin 1 protein		
Variable	All Cases	Low Expression	High Expression	*P* Value*
Age at surgery (years)				0.502
≤50.8^†^	83	44 (53.0%)	39 (47.0%)	
>50.8	86	50 (58.1%)	36 (41.9%)	
Histological type				0.063
Serous	113	65 (57.5%)	48 (42.5%)	
Mucinous	21	8 (38.1%)	13 (61.9%)	
Endometrioid	7	2 (28.6%)	5 (71.4%)	
Clear cell	7	3 (42.9%)	4 (57.1%)	
Undifferentiated	21	16 (76.2%)	5 (23.8%)	
Histological grade (Silveberg)				0.002
G1	29	8 (27.6%)	21 (72.4%)	
G2	100	59 (59.0%)	41 (41.0%)	
G3	40	27 (67.5%)	13 (32.5%)	
pT status				0.037
pT1	47	19 (40.4%)	28 (59.6%)	
pT2	32	18 (56.3%)	14 (43.8%)	
pT3	90	57 (63.3%)	33 (36.7%)	
pN status				0.005
pN0	83	37 (44.6%)	46 (55.4%)	
pN1	86	57 (66.3%)	29 (33.7%)	
pM status				0.001
pMX	146	74 (50.7%)	72 (49.3%)	
pM1	23	20 (87.0%)	3 (13.0%)	
FIGO stage				0.000
I	30	8 (26.7%)	22 (73.3%)	
II	20	8 (40.0%)	12 (60.0%)	
III	96	58 (60.4%)	38 (39.6%)	
IV	23	30 (87.0%)	3 (13.0%)	
Bcl-xL expression				0.001
Low	58	22 (37.9%)	36 (62.1%)	
High	111	72 (64.9%)	39 (35.1%)	

* Chi-square test; ^†^Mean age; FIGO indicates International Federation of Gynecology and Obstetrics.

### Western blotting

Equal amount of tissue lysates were resolved by SDS-polyacrylamide gel electrophoresis (PAGE) and electrotransferred on a polyvinylidene difluoride (PVDF) membrane (Pall Corp., Port Washington, NY) followed by incubating with primary rabbit monoclonal antibodies against human Beclin 1 (Abcam, Cambridge, MA, 1∶1,000 dilution) and Bcl-xL (Cell Signaling, Danvers, MA, 1∶1,000 dilution). The immunoreactive signals were detected with enhanced chemiluminescence kit (Amersham Biosciences, Uppsala, Sweden) according to the manufacturer's instructions.

### Tissue microarray (TMA) and immunohistochemistry (IHC)

Tissue microarray was constructed in accordance with a method described previously [29]. Triplicate 0.6 mm diameter cylinders were punched from representative areas of an individual donor tissue block, and re-embedded into a recipient paraffin block in a defined position, using a tissue arraying instrument (Beecher Instruments, Silver Spring, MD).

The TMA block was cut into 5 µm sections and processed for IHC according to the previously-described protocol [30], [31]. TMA slides were incubated respectively with anti-Beclin 1 (Abcam, Cambridge, MA, 1∶100 dilution) and anti-Bcl-xl (Cell Signaling, Danvers, MA, 1∶100 dilution), and stored overnight at 4°C. Immunostaining was performed using the Envision System with diaminobenzidine (Dako, Glostrup, Denmark). A negative control was obtained by replacing the primary antibody with a normal rabbit IgG.

### IHC evaluation

Immunoreactivity for Beclin 1 and Bcl-xl protein was evaluated in semi-quantitative method as described previously [32]. Each TMA spot was assigned an intensity score from 0–3 (I0, I1–3) and proportion of tumor cells for that intensity over the total number of tumor cells was recorded as 5% increments from a range of 0–100 (P0, P1–3). A final H score (range 0–300) was achieved by adding the sum of scores obtained for each intensity and proportion of area stained (H score = I1XP1+I2XP2+I3XP3).

### Selection of cutpoint score

X-tile plots were generated for assessment of Beclin 1 and Bcl-xL expressions and optimization of cutpoint based on outcome, as described in our previous study [33]. X-tile program divided the cohort randomly into a matched training and validation set as a method for selecting optimal cutpoint, respectively. Statistical significance was assessed by using the cut-off score derived from a training set to parse a separate validation set, using a standard log-rank method, with *P* values obtained from a lookup table. The X-tile plots allowed determination of an optimal cut-off value while correcting for the use of minimum *P* statistics by Miller-Siegmund *P*-value correction [34].

### Statistical analysis

For survival analysis, the optimal cutpoints for the IHC expression were performed using X-tile software version 3.6.1. Monte Carlo simulations were used to adjust for multiple looks in optimal cutoff selection [34]. Receiver operating characteristic (ROC) curve analysis was used to assess the predictive value of the clinicopathologic features. Correlations between variables, ROC curve analysis, univariate survival analysis and multiple Cox proportional hazards regression were performed using SPSS statistical software package (SPSS Standard version 13.0, SPSS Inc.). A significant difference was considered if the *P* value from a two-tailed test was less than 0.05.

## Results

### The expression level of Beclin 1 in ovarian carcinoma and adjacent normal ovarian tissues by Western blotting assay

**Figure 1:**
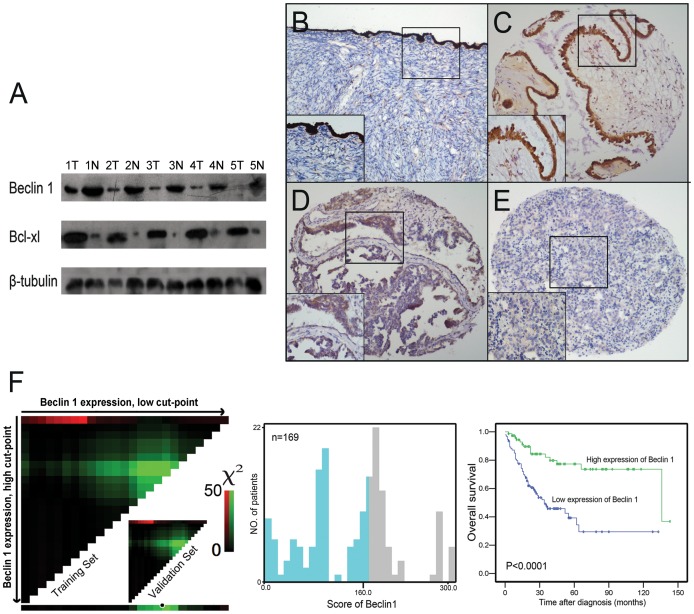
Beclin 1 and Bcl-xL expressions in ovarian tissues and X-tile plots of Beclin 1 expression in ovarian carcinomas. (A) Western blotting analysis of Beclin 1 and Bcl-xL expressions in ovarian carcinoma tissues (T) and adjacent normal ovarian tissues (N). (B) High expression of Beclin 1 was observed in epithelia cells of normal ovary by immunohistochemistry. (C) Highly-expressed Beclin 1 was examined in a cystadenoma case 12. (D) An ovarian borderline tumor (Case 18) showed immunoreactivity of Beclin 1 mainly in cytoplasm. (E) Low expression of Beclin 1 was detected in an ovarian carcinoma case (Case 79). Representative sites in tissue microarray with low (×100) and high (inset, ×400) magnification were shown. (F) X-tile analysis was employed to determine the cutpoint for Beclin 1 expression, by equally dividing the total patients into training and validation subsets. X-tile plots of training sets were displayed in the left panels, with matched validation sets in the smaller inset. The plot showed the χ^2^ log-rank values generated when dividing the cohort into two populations. The cutpoint (H score = 160) highlighted by the black/white circle in the horizontal axis (left panel) was demonstrated on a histogram of the entire cohort (middle panel), and a Kaplan-Meier plot (right panel).

In this study, the protein expression of Beclin 1 was first examined by Western blotting in 5 pairs of primary ovarian carcinoma and adjacent normal ovarian tissues. An apparent decrease in protein expression of Beclin 1 was detected in ovarian carcinoma tissues compared to adjacent ovarian tissues (Figure 1A).

### Beclin 1 expression in ovarian tissues examined by IHC

Immunoreactivity for Beclin 1 was examined primarily in the cytoplasm of ovarian surface epithelial and tumor cells (Figure 1B∼1E). Beclin1 and Bcl-xL expressions could be evaluated successfully and simultaneously in 230 epithelial ovarian tumors (including 26 cystadenomas, 35 borderline tumors and 169 invasive carcinomas) of the TMA constructed previously and in 12 normal ovaries. The 19 cases of non-informative TMA sample included unrepresentative areas, samples with too few tumor cells (<300 cells per case) and lost samples. According to X-tile program, H score for Beclin 1 expression above the cutpoint value 160 was defined as high expression (Figure 1F). Similarly, X-tile plots indicated that a score of 100 was also the cutpoint to distinguish the cancer patients as high or low Bcl-xL expression (data not shown). In this study, decreased expression of Beclin 1 was examined in 94/169 (55.6%) of ovarian carcinomas. The increasing frequency of decreased expression of Beclin 1 in normal ovarian tissues (8.3%), benign cystadenomas (15.4%), borderline tumors (20.0%), and ovarian carcinomas (55.6%) was statistically significant (*P*<0.0001, Table S1). In addition, low-expressed Beclin 1 was closely linked to tumor poorer differentiation (Figure S1), later pT stage, lymph node metastasis, distant metastasis and advanced FIGO stage (*P*<0.05, Table 1).

### Relationship between clinicopathologic variables, Beclin 1 expression and ovarian carcinoma patients' survival

**Figure 2:**
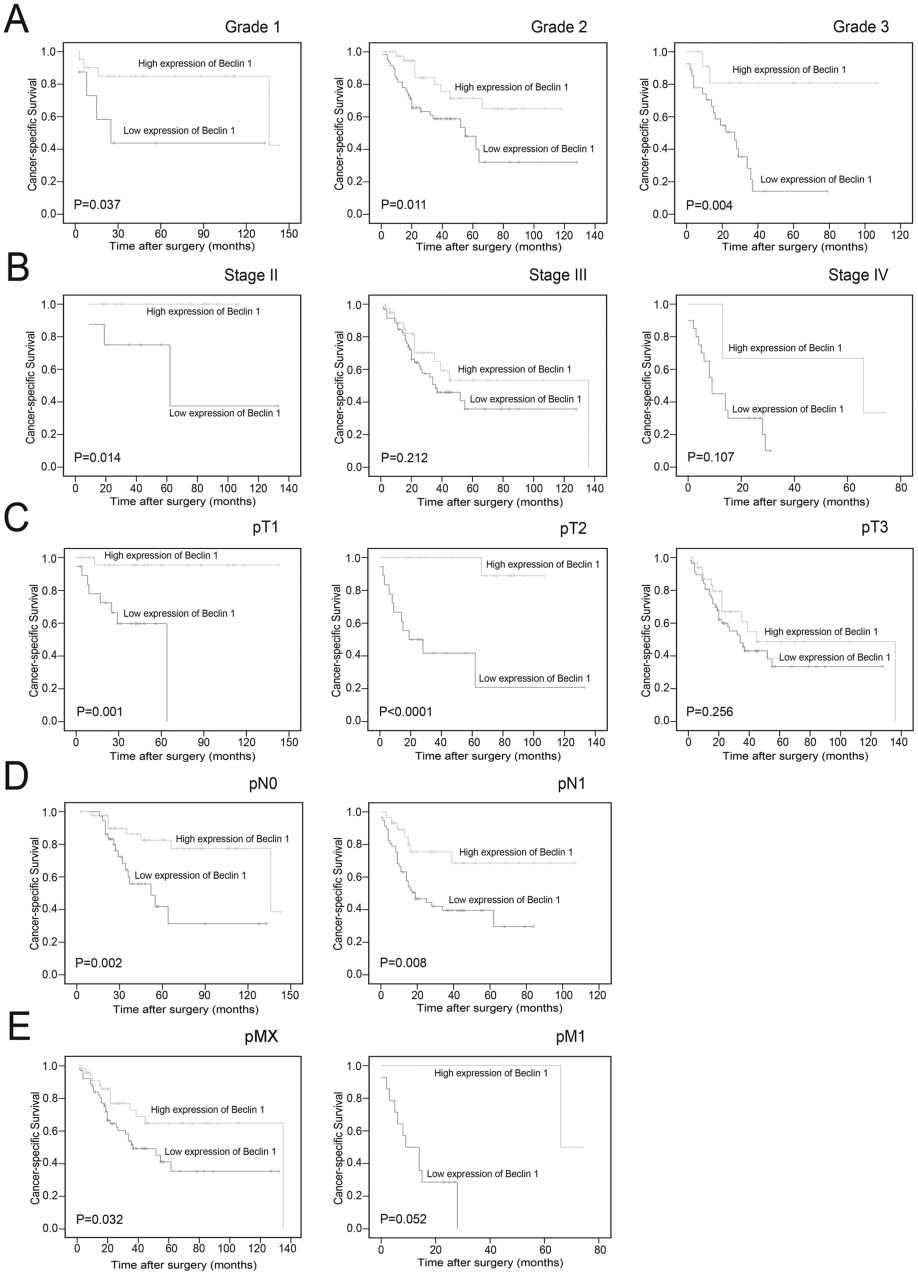
Kaplan-Meier survival analysis of Beclin 1 expression in subsets of patients with ovarian carcinoma (log-rank test). (A) Survival analysis of Beclin1 expression in subsets of different grade patients: left panel, grade 1; middle panel, grade 2; right panel, grade 3. (B) Survival analysis of Beclin1 expression in subsets of different stage patients: left panel, stage II; middle panel, stage III; right panel, stage IV. (C) Survival analysis of Beclin1 expression in subsets of different pathologic T stage patients: left panel, pT1; middle panel, pT2; right panel, pT3. (D) Survival analysis of Beclin1 expression in subsets of different pathologic N stage patients: left panel, pN0; right panel, pN1. (E) Survival analysis of Beclin1 expression in subsets of different pathologic M stage patients: left panel, pMX; right panel, pM1.

Kaplan–Meier analysis evaluated significant impact of well-known clinicopathologic prognostic variables, such as histological grade (*P* = 0.020), pT status (*P* = 0.005), pN status (*P*<0.0001), pM status (*P*<0.0001) and FIGO stage (*P*<0.0001) on patients' survival (Table 2). Assessment of patients survival revealed that decreased expression of Beclin 1 was closely associated with poor disease-specific survival (*P*<0.0001, Figure 1F), and the mean survival time for patients with tumor having low-expressed Beclin 1 was 57.8 months compared to 110.2 months for patients with tumor having highly-expressed Beclin 1 (Table 2). Moreover, survival analysis was performed with regards to Beclin 1 expression in subsets of patients with different histological grades, pT/pN/pM status and FIGO stages. The results demonstrated that low-expressed Beclin 1 was as well an adverse prognostic factor in ovarian carcinoma patients having tumor in grade 1 (*P* = 0.037), grade 2 (*P* = 0.011), grade 3 (*P* = 0.004; Figure 2A), FIGO stage II (*P* = 0.014, Figure 2B), pT1 (*P* = 0.001), pT2 (*P*<0.0001, Figure 2C), pN0 (*P* = 0.002, Figure 2D), pN1 (*P* = 0.008, Figure 2D), pMX (*P* = 0.032, Figure 2E).

**Table 2:** Univariate and Multivariate Analysis of Different Prognostic Features in 169 Patients with Ovarian Carcinoma.

Variable	Univariate Analysis*				Multivariate Analysis^†^	
	All Cases	Mean Survival (Months)	Median Survival (Months)	*P* Value	HR (95% CI)	*P* Value
Age at surgery (years)				0.797		
≤50.8^‡^	83	80.4	66.0			
>50.8	86	84.6	NR			
Histological type				0.421		
Serous	113	70.5	72.0			
Mucinous	21	82.6	81.0			
Endometrioid	7	132.4	NR			
Clear cell	7	102.8	NR			
Undifferentiated	21	35.2	NR			
Histological grade (Silveberg)				0.020	0.987 (0.629–1.548)	0.955
G1	29	105.6	136.0			
G2	100	77.6	66.0			
G3	40	50.0	29.0			
pT status				0.005	1.246 (0.804–1.933)	0.325
pT1	47	110.2	NR			
pT2	32	84.9	NR			
pT3	90	66.6	37.0			
pN status				0.000	2.121 (1.219–3.689)	0.008
pN0	83	96.6	136.0			
pN1	86	55.7	39.0			
pM status				0.000	1.228 (0.335–4.503)	0.757
pMX	146	91.1	136.0			
pM1	23	23.4	13.0			
FIGO stage				0.000	2.965 (1.213–7.244)	0.017
I	30	134.2	NR			
II	20	113.7	NR			
III	96	71.2	45.0			
IV	23	23.4	13.0			
Beclin 1expression				0.000	0.489 (0.273–0.909)	0.013
Low	94	57.8	34.0			
High	75	110.2	136.0			
Bcl-xl expression				0.028	1.894 (0.859–3.556)	0.147
Low	58	84.2	NR			
High	111	74.5	34.0			

* Log-rank test; ^†^Cox regression model; ^‡^Mean age; HR indicates hazards ratio; CI indicates confidence interval; NR indicates not reached; FIGO indicates International Federation of Gynecology and Obstetrics.

### Independent prognostic factors of ovarian carcinoma: multivariate survival analysis

Multivariate Cox proportional hazard regression analysis was employed to identify the independent value of each variable for predicting patients overall survival (Table 2). Expression of Beclin 1 and clinicopathologic characteristics (including histological grade, FIGO stage, pT stage, pN stage and pM stage) that showed significant effect on overall survival by univariate analysis were included in multivariate analysis (Table 2). As anticipated, low expression of Beclin 1 was identified as an independent risk factor of patients poor survival (relative risk: 0.489, CI: 0.273–0.909, *P* = 0.013). With regard to other features, only pN stage (*P* = 0.008, Table 2) and FIGO stage (*P* = 0.017, Table 2) were shown to be independent prognostic predictors for patients overall survival.

### Correlation between expression of Beclin 1 and Bcl-xL in ovarian carcinoma tissues

**Figure 3:**
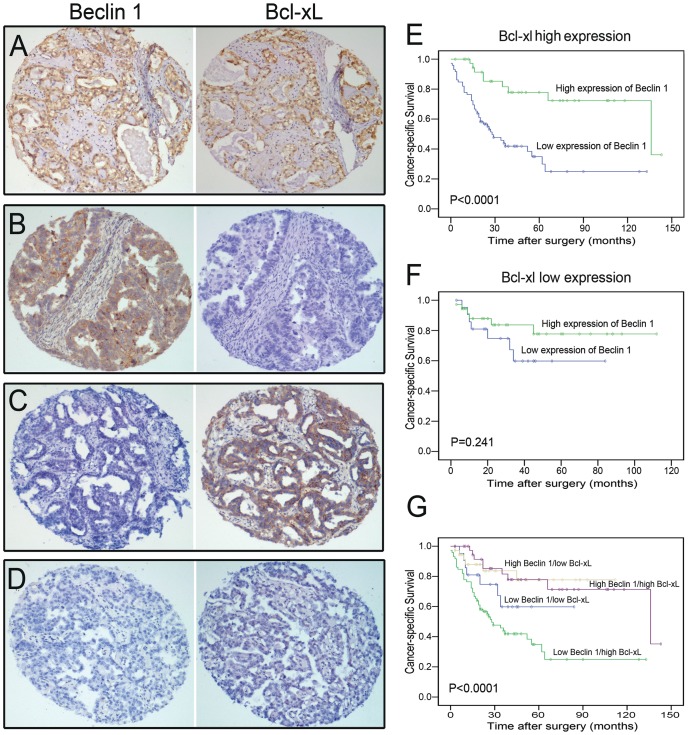
The expression patterns of Beclin 1and Bcl-xL in ovarian carcinoma and Kaplan-Meier survival analysis. Consecutive sections were used for immunohistochemical study for Beclin 1 and Bcl-xL. (A) High Beclin 1/high Bcl-xL case (Case 9). (B) High Beclin 1/low Bcl-xL case (Case 17). (C) Low Beclin 1/high Bcl-xL (Case 42). (D) Low Beclin 1/low Bcl-xL case (Case 63, Magnification, ×100). (E) Kaplan-Meier analysis of overall survival (OS) for Beclin 1 expression in Bcl-xL–high population. (F) Kaplan-Meier analysis of OS for Beclin 1 expression in Bcl-xL–negative population. (G) Combined analysis of Beclin 1 and Bcl-xL expression in the prognostic value of patients with ovarian cancer (Log-rank test).

Our western blotting assay showed an inverse correlation between protein expression levels of Beclin 1 and Bcl-xL in ovarian tissues (Figure 1A). By utilizing the criterion described before, high expression of Bcl-xL was detected in 111/169 (65.7%) of our ovarian carcinomas by IHC. Further correlation analysis showed a significant inverse correlation between Beclin 1 and Bcl-xL expressions in our ovarian carcinoma cohort (*P* = 0.001, Fishers exact test; Table 1; Figure 3A–3D).

### Combined decreased expression of Beclin 1 with high expression of Bcl-xL was correlated with poorer prognosis in ovarian carcinoma patients

To further determine whether altered expression of Bcl-xL influences the autophagy-related prognosis, we divided the ovarian carcinoma cases into low Bcl-xL expression (Bcl-xL^−^) and high Bcl-xL expression (Bcl-xL^+^) sets according to the cutpoint. When all cases of ovarian carcinoma were stratified by Bcl-xL expression, we found that the prognosis of patients with low Beclin 1 expression was significantly poorer than that with high Beclin 1 expression in the Bcl-xL^+^ group (*P*<0.0001, Figure 3E). However, in the Bcl-xL^−^ group, no significant difference in survival times was found between patients with low and high expression of Beclin 1 (*P* = 0.241, Figure 3F). In combined analysis of Beclin 1 and Bcl-xL expression, we further evaluated that the low Beclin 1/high Bcl-xL group had the worst survival (mean survival time, 52.2 months), the low Beclin 1/low Bcl-xL and the high Beclin 1/high Bcl-xL groups moderate survival (58.1 and 91.5 months), and the high Beclin 1/low Bcl-xL group the best survival (109.9 months, *P*<0.0001, Figure 3G).

**Figure 4:**
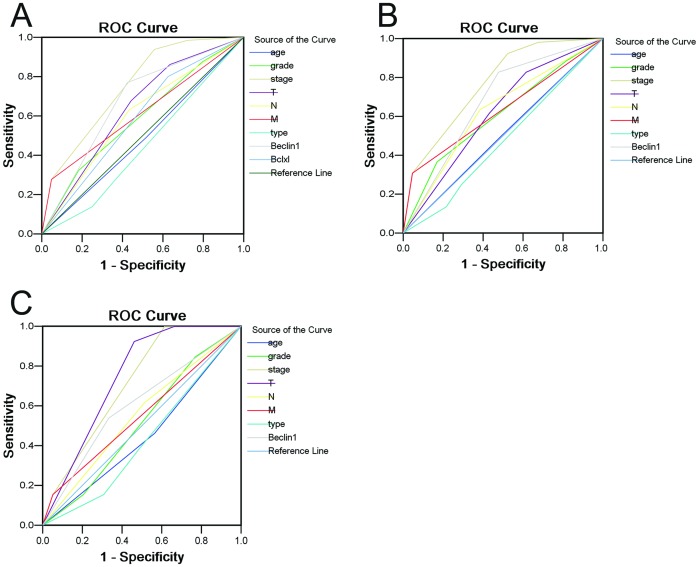
Receive operating characteristic curve analysis for several clinicopathological features and Beclin 1 expression was used to evaluate the survival status. (A) Age [area under curve (AUC) = 0.487, *P* = 0.769], histological grade (AUC = 0.588, *P* = 0.054), FIGO stage (AUC = 0.750, *P*<0.0001), pT status (AUC = 0.638, *P* = 0.003), pN status (AUC = 0.599, *P* = 0.031), pM status (AUC = 0.614, *P* = 0.012), histological type (AUC = 0.446, *P* = 0.242),Beclin 1 expression (AUC = 0.673, *P*<0.0001) and Bcl-xL (AUC = 0.588, *P* = 0.056) implied statistical associations with the survival in whole study population. (B) Age (AUC = 0.504, *P* = 0.943), histological grade (AUC = 0.609, *P* = 0.044), FIGO stage (AUC = 0.767, *P*<0.0001), pT status (AUC = 0.617, *P* = 0.030), pN status (AUC = 0.625, *P* = 0.020), pM status (AUC = 0.631, *P* = 0.015), histological type (AUC = 0.472, *P* = 0.598),and Beclin 1 expression (AUC = 0.675, *P*<0.0001) were applied to test the survival status in highly-expressed Bcl-xL population. (C) Age (AUC = 0.449, *P* = 0.583), histological grade (AUC = 0.511, *P* = 0.907), FIGO stage (AUC = 0.714, *P* = 0.022), pT status (AUC = 0.744, *P* = 0.009), pN status (AUC = 0.551, *P* = 0.583), pM status (AUC = 0.551, *P* = 0.583), histological type (AUC = 0.427, *P* = 0.434),and Beclin 1 expression (AUC = 0.603, *P* = 0.272) were employed to evaluate the survival in low-expressed Bcl-xL population.

To assess prognostic values of Beclin 1 expression in total ovarian carcinoma patients, in highly-expressed Bcl-xL and in low-expressed Bcl-xL groups, ROC curves were plotted to evaluate the patients' survival status. ROC curve analysis confirmed the promising predictive significance of Beclin 1 with regard to specific survival in all carcinoma patients [area under curve (AUC) = 0.673, Figure 4A). When further analyzed in Bcl-xL^+^ group, Beclin 1 was evaluated as well an encouraging prognostic predictor for ovarian carcinoma patients survival (AUC = 0.675, *P*<0.0001, Figure 4B). In contrast, there was no statistical significance in the Bcl-xL^−^ group (AUC = 0.603, *P* = 0.272, Figure 4C).

## Discussion

Autophagy occupies the center of a complex network of cellular responses to stressors in eukaryotic cells. It has been suggested that autophagy is involved in multiple pathological processes, including human cancers. Oncogenesis and tumor survival are influenced by disturbances of the molecular machinery that controls autophagy [35]. *Beclin 1*, one essential component of autophagy, has been identified as a haplo-insufficient tumor-suppressor gene. Qu et al [20] reported that monoallelic deletion of *Beclin 1* could lead to tumorigenesis by using targeted mutant mouse model. In addition, heterozygous disruption of *Beclin 1* also increased the frequency of spontaneous malignant diseases and accelerated the development of hepatitis B virus–induced premalignant lesions. Thus, inactivation of autophagic genes such as *Beclin 1* may contribute to the development of human cancers [36].

In the present study, we investigated the expression patterns of Beclin 1 and Bcl-xL, by Western blotting and IHC, using fresh ovarian tissues and a TMA containing a series of benign, borderline and malignant epithelial ovarian tumors. Western blotting assay revealed that down-regulation of Beclin 1 was detected in ovarian carcinoma tissues, when compared with their adjacent normal ovarian tissues. Moreover, our IHC results demonstrated that an increasing frequency of decreased expression of Beclin 1 was observed from benign (cystadenoma) to borderline tumors, and to malignant carcinomas. It was reported that in other types of human cancer, such as breast, prostate, liver and nasopharyngeal cancers, down-regulated expression of Beclin 1 was also frequently observed [6], [21], [25], [27], [37]. These data provided evidence that the defective expression of Beclin 1 might play an important role in tumorigenic process of different human cancers, including ovarian carcinoma.

Further correlation analyses evaluated that low expression of Beclin 1 in our ovarian carcinoma cohort was positively correlated with an ascending histological grade, late pT stage, lymph node metastasis, distant metastasis and/or advanced FIGO stage. These findings suggested that decreased expression of Beclin 1 in ovarian carcinomas may contribute to an increased malignant phenotype. Similar results were also found in other human malignancies, such as esophageal, hepatocellular and colon cancers and high-grade glioma, in which down-regulated expression of Beclin 1 was frequently observed in more aggressive tumor subgroups and had a worse prognosis [37], [38], [39], [40]. In this study, we evaluated that decreased expression of Beclin 1 in ovarian carcinoma was as well a strong and independent predictor of short patient survival. More importantly, stratified survival analysis of histological grade, pT/pN/pM status and clinical stage showed that Beclin 1 expression was also linked closely to survival of different subsets of patients with ovarian carcinoma. Thus, Beclin 1 expression appears to have the potential to predict ovarian carcinoma patient clinical outcome. The examination of Beclin 1 expression, by IHC, therefore, could be used as an additional effective tool in identifying those ovarian carcinoma patients at increased risk of tumor invasion and/or progression.

It has been reported that the tumor suppressor Beclin 1 may coordinate both apoptosis and autophagy through direct interaction with antiapoptotic family protein Bcl-xL [9], [18]. Beclin1 contains a conserved BH3 domain and determined the crystal structure of the Beclin 1 BH3 peptide in complex with Bcl-xL [41]. In addition, Bcl-xL has recently been identified as a mitochondrial ARF-binding protein, which normally protects cells from autophagy by inhibiting the Beclin-1/Vps34 complex [42]. Thus, in this study, the expression dynamics of Bcl-xL and its correlation with Beclin 1 in ovarian carcinomas were subsequently investigated. Our results showed that high expression of Bcl-xL was frequently examined by IHC in ovarian carcinomas, and it was associated closely with shortened survival times of the patients. Further correlation analysis demonstrated that Beclin 1 expression was significantly inversely correlated with Bcl-xL expression in our ovarian carcinoma cohort. To determine if altered expression of Bcl-xL influences the Beclin 1-related prognosis, we evaluated the ovarian carcinoma patients' survival after stratification by Bcl-xL expression. We found that low expression of Beclin 1 was, as well, closely associated with poor prognosis of ovarian carcinoma in the Bcl-xL^+^ group, but not in the Bcl-xL^−^ group. This observation was confirmed by ROC curve analysis, in which Beclin 1 was evaluated as a promising prognostic predictor for patient survival status in the Bcl-xL^+^ group. Furthermore, combined analysis of Beclin 1 and Bcl-xL expression, the low Beclin 1/high Bcl-xL group suffered from the lowest survival rate compared to other groups. Thus, we speculated that defect in Bcl-xL expression may influence the Beclin 1-related prognosis in ovarian carcinoma, and that coordination of autophagy and apoptosis may play a more significant role in the tumorigenesis and/or progression of this human malignancy.

In summary, in this study, we describe the expression status of Beclin 1 in normal human ovary, benign, borderline and malignant epithelial ovarian tumor tissues. Our results provide a basis for the concept that decreased expression of Beclin 1 may represent an acquired malignant phenotypic feature of ovarian carcinoma cells. In addition, our study introduces Beclin 1 protein expression as a new independent prognostic marker in ovarian carcinomas, and more importantly, decreased expression of Beclin 1 synergized with altered Bcl-xL expression in tumor cells predicts poorer outcome of the cancer for the individual patient.

## Supporting Information

Figure S1 **The altered expression levels of Beclin 1 in ovarian carcinoma tissues by immunohistochemistry.** (A) A well-differentiated (Grade 1) ovarian carcinoma (Case 53) showed high expression of Beclin 1. (B) A poor-differentiated (Grade 3) ovarian cancer (Case 136) was examined negative expression of Beclin 1. Left panels, hematoxylin-eosin staining; right panels, immunohistochemical staining. Representative sites in ovarian carcinoma tissue with low (×100) and high (inset, ×400) magnification were shown. (C) Relative Beclin 1 protein levels in nonmetastatic and metastatic primary cancer tissues were detected by Western blotting assay.
(TIF)

Table S1 **Expression Patterns of Beclin 1 Protein in Normal Ovaries and in a Series of Epithelial Ovarian Tumours.**
(DOC)
