# Supplementary figures and images for: Decreased Expression of Beclin 1 Correlates Closely with Bcl-xL Expression and Poor Prognosis of Ovarian Carcinoma
Source: PLoS One. 2013 Apr 3;8(4):e60516. doi: 10.1371/journal.pone.0060516 (PMC3616009; doi:10.1371/journal.pone.0060516)

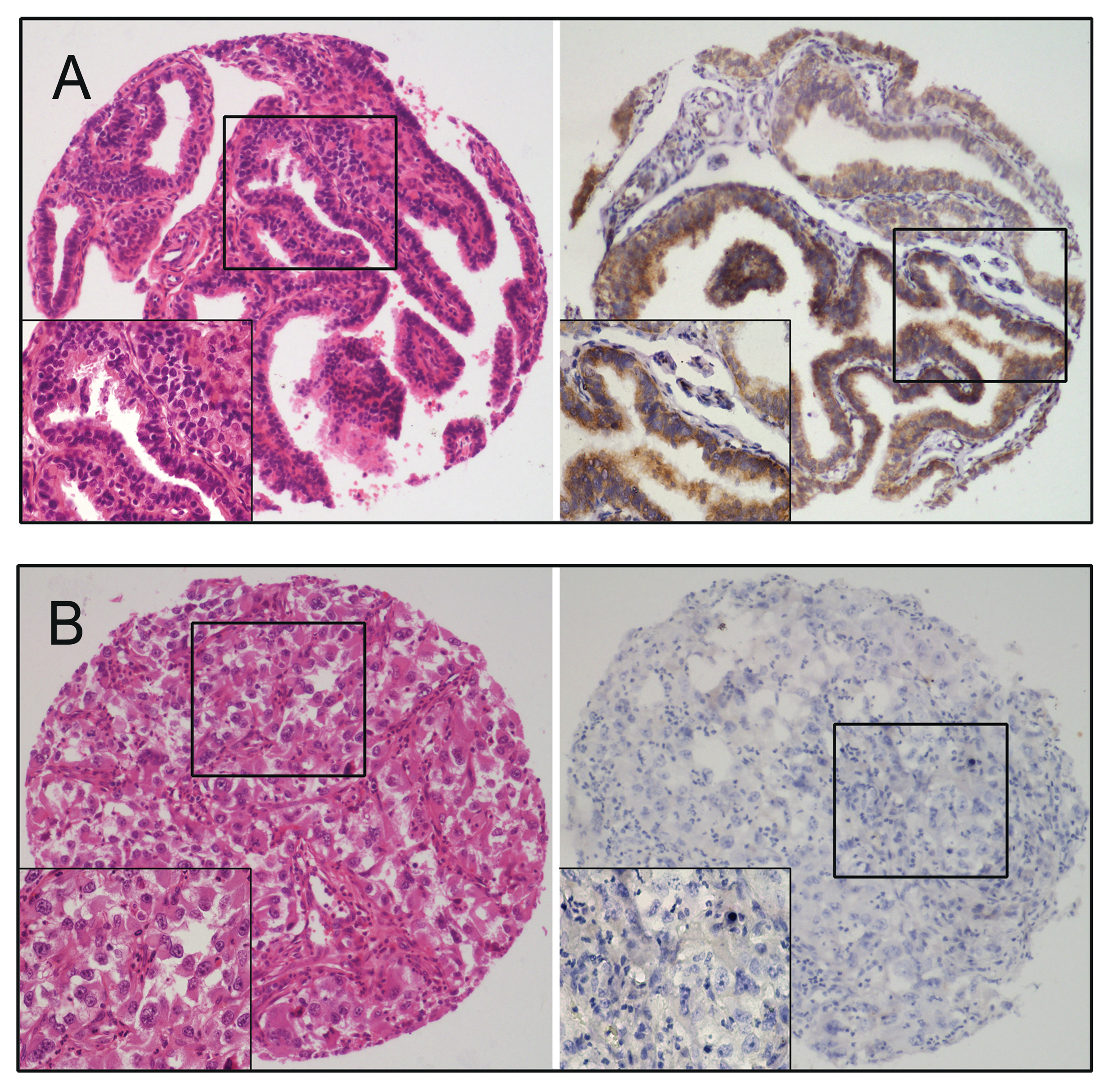

Supplement: Figure S1 — The altered expression levels of Beclin 1 in ovarian carcinoma tissues by immunohistochemistry. (A) A well-differentiated (Grade 1) ovarian carcinoma (Case 53) showed high expression of Beclin 1. (B) A poor-differentiated (Grade 3) ovarian cancer (Case 136) was examined negative expression of Beclin 1. Left panels, hematoxylin-eosin staining; right panels, immunohistochemical staining. Representative sites in ovarian carcinoma tissue with low (×100) and high (inset, ×400) magnification were shown. (C) Relative Beclin 1 protein levels in nonmetastatic and metastatic primary cancer tissues were detected by Western blotting assay. (TIF) [file pone.0060516.s001.tif]
