# Supplementary material for: Decreased Expression of Beclin 1 Correlates Closely with Bcl-xL Expression and Poor Prognosis of Ovarian Carcinoma
Source: PLoS One. 2013 Apr 3;8(4):e60516. doi: 10.1371/journal.pone.0060516 (PMC3616009; doi:10.1371/journal.pone.0060516)
Supplement: Table S1 — Expression Patterns of Beclin 1 Protein in Normal Ovaries and in a Series of Epithelial Ovarian Tumours. (DOC) [file pone.0060516.s002.doc]

**Table S1.** Expression Patterns of Beclin 1 Protein in Normal Ovaries and in a Series of Epithelial Ovarian Tumours^*^

|  |  | **Beclin 1 Protein** | |
| --- | --- | --- | --- |
|  | **All Cases** | **Low Expression** | **High Expression** |
| **Normal ovaries** | 12 | 1 (8.3%) | 11 (91.7%) |
| **Cystadenomas** | 26 | 4 (15.4%) | 22 (84.6%) |
| **Borderline tumors** | 35 | 7 (20.0%) | 28 (80.0%) |
| **Invasive carcinomas** | 169 | 94 (55.6%) | 75 (44.4%) |

^*^Values are n (%). A significant decreasing frequency of high expression of Beclin 1 was examined in cystadenomas, borderline tumors and invasive carcinomas (*P*<0.0001, Chi-Square Test).
